# Supplementary material for: Impact of ANCA-Associated Vasculitis on Outcomes of Hospitalizations for Goodpasture’s Syndrome in the United States: Nationwide Inpatient Sample 2003–2014
Source: Medicina (Kaunas). 2020 Mar 1;56(3):103. doi: 10.3390/medicina56030103 (PMC7142422; doi:10.3390/medicina56030103)
Supplement: Supplementary file 1 [file medicina-56-00103-s001.pdf]

Table S1 ICD-9 code for treatments and outcomes

	Description	ICD-9CM
Organ Failure		
Respiratory	Acute respiratory failure	518.81
	Other pulmonary insufficiency, not elsewhere classified. Includes - acute respiratory distress, acute respiratory insufficiency, adult respiratory distress syndrome NEC	518.82
	Acute respiratory distress syndrome after shock or trauma	518.85
	Respiratory distress NOS	786.09
	Respiratory arrest	799.1
	Ventilator management	96.7, 96.70, 96.71, 96.72
Cardiovascular	Shock without mention of trauma	785.5
	Shock unspecified	785.50
	Other shock without trauma (includes hypovolemic Shock)	785.59
	Cardiogenic shock	785.51
	Septic shock	785.52
	Hypotension NOS	458.8, 458.9, 796.3
Renal	Acute kidney injury	584, 584.5, 584.6, 584.7, 584.8, 584.9
Hepatic	Acute hepatic failure or necrosis	570
	Hepatic encephalopathy	572.2
	Hepatitis unspecified	573.3
	Hepatic infarction	573.4
Hematologic	Defibrination syndrome	286.6
	Acquired coagulation factor deficiency	286.7
	Other coagulation defect	286.9
	Thrombocytopenia - secondary or unspecified	287.49, 287.5
Metabolic	Acidosis – metabolic or lactic	276.2
Neurologic	Transient organic psychotic conditions	293, 293.0, 293.1, 293.8, 293.81, 293.82, 293.83, 293.84, 293.89, 293.9
	Anoxic brain injury	348.1
	Acute encephalopathy	348.3, 348.30, 348.31, 348.39
	Coma	780.01
	Altered consciousness - unspecified	780.09
	Electroencephalogram	89.14
Sepsis	Sepsis	038.0, 038.10, 038.11, 038.19, 038.2, 038.3, 038.4, 038.40, 038.41, 038.42, 038.43, 038.44, 038.49, 038.8, 038.9, 790.7, 117.9, 112.5, 115.04, 115.14,

		115.94, 112.81, 112.83, 003.1, 003.21, 036.2, 036.3, 036.0, 036.1, 036.42, 020.2, 022.3, 098.89, 098.84, 098.82, 995.92, 785.52
Procedure		
Plasmapheresis		99.71, 99.76, 99.79
Invasive mechanical ventilation		96.7, 96.70, 96.71, 96.72
Non-invasive mechanical ventilation		93.90
Renal replacement therapy		39.95, 54.98
